# Treatment of bronchial airway obstruction using a rotating tip microdebrider: a case report

**DOI:** 10.1186/1749-8090-2-16

**Published:** 2007-03-26

**Authors:** Marcus P Kennedy, Rodolfo C Morice, Carlos A Jimenez, George A Eapen

**Affiliations:** 1 Department of Interventional Pulmonology, University of Texas MD Anderson Cancer Center, Houston, Texas, USA

## Abstract

**Background:**

Central airway obstruction is a common complication of lung cancer. The microdebrider is a new device available for treatment of central airway obstruction.

**Case Description:**

We report a case a 59-yr-old male with T3N2M1 non-small cell lung cancer with malignant distal left mainstem obstruction treated successfully with a novel elongated rotating tip microdebrider via rigid bronchoscopy with sufficient length to reach distal bronchial lesions.

**Discussion and Conclusion:**

The microdebrider is an excellent addition to the spectrum of interventions available for the management of central airway obstruction with advantages including accuracy and immediate removal of debris without a need for separate suctioning or limitation in oxygenation.

## Background

The microdebrider is a recent addition to the armeratarium available to the interventional bronchoscopist for central airway occlusion, following the successful application of laryngeal microdebriders for over a decade [1-3]. However, limitations of the microdebrider include maneuverability and length (37 cms) which limits use to the trachea and proximal main-stem bronchi. We report a case of malignant distal left main-stem obstruction treated with a new elongated rotating tip microdebrider (45 cms) via rigid bronchoscopy with sufficient length to reach distal bronchial lesions.

## Case Description

A 53-yr-old male with T3N2M1 non-small cell left upper lobe lung cancer with bilateral brain metastases diagnosed one-month prior was referred for management of dyspnea and hemoptysis. The patient had noted a dramatic increase in dyspnea over the prior 3 days. Past medical history was unremarkable and he had a negative smoking history.

**Figure 1:**
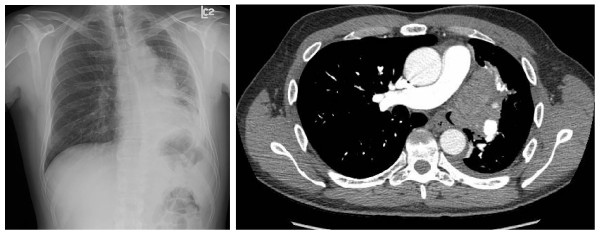
**a **Posterior anterior chest x-ray of 57-yr-old male with T3N2M1 non-small cell lung cancer demonstrates a left hilar mass with loss of volume and/or post obstructive pneumonia in the left lower lobe. **b **Axial computed tomography chest angiogram demonstrates total occlusion of the left mainstem bronchus by a tumor invading from the left hilum.

Physical exam revealed reduced breath sounds in the left lower zone. Chest x-ray demonstrated a left hilar mass with loss of volume and post obstructive pneumonia in the left lower lobe (Figure 1a). Axial Computed tomography (CT) angiogram of the chest also revealed multiple segmental and subsegmental pulmonary emboli and the patient was admitted for anticoagulation with low molecular weight heparin (enoxaparin). The CT study also confirmed total occlusion of the left mainstem bronchus by a tumor invading from the left hilum (Figure 1b).

**Figure 2:**
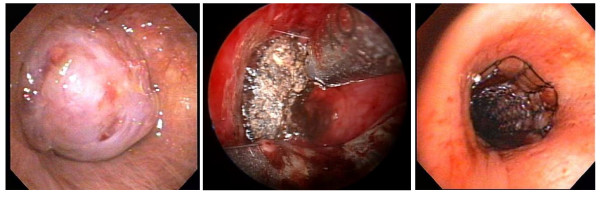
**a **Videobronchoscopy image demonstrates distal left mainstem bronchus occlusion. **b **Videobronchoscopy image demonstrates debridement of malignant distal mainstem occlusion with the rotating tip tracheal microdebrider after argon plasma coagulation. **c **Videobronchoscopy image demonstrates a nitinol self-expandable metal stent in the previously occluded left mainstem bronchus.

**Figure 3:**
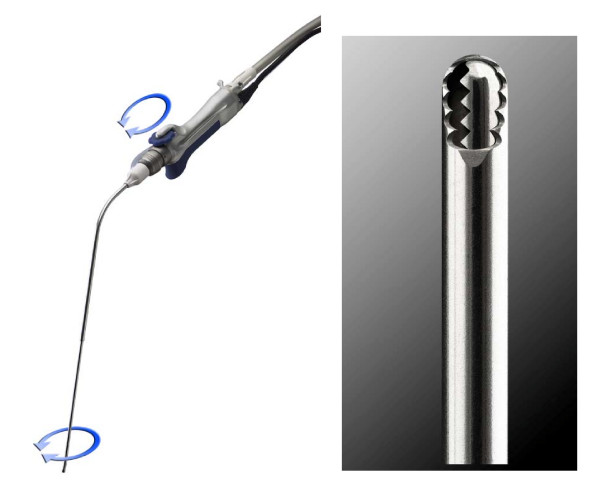
The rotating tip tracheal microdebrider.

**Figure 4:**
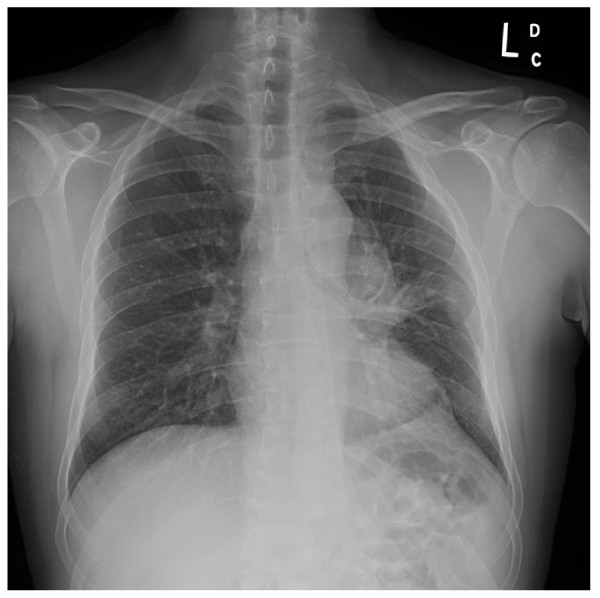
Posterior anterior chest x-ray demonstrates satisfactory stent placement with re-expansion of the left lower lobe.

After two days of anticoagulation, rigid bronchoscopy was performed using an Efer-Dumon rigid bronchoscope. Normal vocal cords, trachea, carina and right bronchial tree were confirmed. A large endobronchial lesion with complete occlusion of the left mainstem bronchus was identified (Figure 2a). After an unsuccessful attempt to pass the lesion using a pediatric video bronchoscope, debulking was attempted using a combination of polypectomy snare and electrocautery. There was still 100% obstruction of the airway from the residual tumor and therefore the ERBE argon plasma electrocoagulator^® ^(APC) was utilized to devitalize the tissue. The devitalized tissue was then debulked using the Straightshot^® ^microdebrider with rotating tip (Figures 2b, 3) and the bevel of the rigid bronchoscope. The length of the microdebrider is 45 cm and outer diameter is 4 mm. The rotating tip is controlled by a fly-wheel on the hand-piece, which allows for better maneuverability and is angled at 12 degrees providing better visualization. The blade was serrated and an oscillator mode was used at 1000–1200 RPMs. In this manner the left main stem was recanalized and the channel was opened into the left lower lobe bronchus. Hemostasis was achieved using a combination of suction and APC with less than 20cc blood loss. Visual examination revealed that there was some degree of extrinsic compression in the left lower lobe bronchus as well; however, the superior segment bronchus and the basal segmental bronchi were noted to be patent. Post procedure residual obstruction in the left main stem bronchus was estimated at less than 20%. Since the left upper lobe had been completely replaced by tumor, a guidewire was then placed into the left lower lobe bronchus and a 4 cm × 14 mm covered nitinol self-expandable metal stent was placed over the guidewire under direct vision into the left lower lobe bronchus. The stent was deployed under direct vision and adjusted in terms of its position using rat-tooth forceps. At the end of optimal positioning, the distal aspect of the stent was noted to be just inside the left lower lobe basal segmental bronchus with the superior segment being ventilated through the uncovered portion of the stent. The proximal aspect of the stent was noted in the left main stem bronchus well above the proximal extent of the tumor (Figure 2c). Follow-up chest x-ray (Figure 4) revealed aeration of the left lower lobe with resolution of dyspnea and hemoptysis.

## Discussion and Evaluation

Approximately 20–30% of patients with lung cancer will develop airway obstruction and therefore the management of airway obstruction is a vital component of the services provided by an interventional pulmonology program [4]. Many modalities are currently available, the majority of which rely on thermal injury to the targeted area. These techniques include electrocautery, argon plasma coagulation, cryotherapy, laser therapy, brachytherapy and photodynamic therapy, each with their own advantages and disadvantages [5]. Disadvantages of thermal modalities as a group include uncontrolled thermal injury to normal tissue with complications such as perforation and fistula, limitation of inspired oxygen concentration because of the risk of endobronchial fire and the need for separate suction devices, which requires intermittent removal of the thermal device from the bronchoscope with prolongation of the procedure.

Microdebriders accomplish debridement using a powered rotating blade coupled with suction allowing rapid removal of debris. To date, microdebriders have been used primarily in sinus surgery and advantages include rapid accurate debridement with maintenance of vision because of instantaneous removal of blood and debris through integrated suction [6]. Subsequently, microdebriders were designed for the management of laryngotracheal carcinoma with successful debridement and avoidance of tracheotomy [2]. Tracheal blades were subsequently designed up to 37 cm in length and allowed access to tracheal lumen and proximal mainstem and a retrospective analysis of 23 patients highlighted successful treatment of tracheal granulation tissue, idiopathic subglottic stenosis and malignant disease [1]. The novel device used in this case report has a number of advantages over prior microdebrider devices. The added length (8 cm) allows access to distal bronchial lesions. The rotating tip allows better maneuverability and precision, reducing the possibility of debriding normal tissue.

It is unclear presently what modality is superior for the treatment of central airway obstruction. Approaches depend on the lesion involved and operator preference and experience. We agree with other authors that microdebridement is useful when precision is essential; especially where high flow oxygen is required [1]. A comparison of microdebrider bronchoscopy to thermal modalities to identify the best strategy for the management of central airway obstruction will be difficult, because of the difficulty of designing blinded randomized controlled trials in this critically ill population. In fact, this case highlights the benefit of using multiple modalities to treat central airway obstruction. The limitations of one modality (bleeding and damage of tissue for pathological inspection with microdebridement) can often be overcome by combining various therapeutic options.

## Conclusion

In conclusion, this case report details the successful management of a distal left mainstem obstructing malignant lesion with a combination of therapies including a novel elongated microdebrider with rotating tip. Prospective trials are required to identify specific indications for this therapy along with long-term outcomes in the management of central airway obstruction.

## Abbreviations

CT = computed tomography

## Competing or Financial interests

The author(s) declare that they have no competing interests.
